# Improving face identity perception in age-related macular degeneration via caricaturing

**DOI:** 10.1038/s41598-018-33543-3

**Published:** 2018-10-12

**Authors:** Jo Lane, Emilie M. F. Rohan, Faran Sabeti, Rohan W. Essex, Ted Maddess, Nick Barnes, Xuming He, Rachel A. Robbins, Tamara Gradden, Elinor McKone

**Affiliations:** 1 Research School of Psychology, and ARC Centre of Excellence in Cognition and its Disorders, The Australian National University, Canberra, ACT Australia; 2 John Curtin School of Medical Research (JCSMR), The Australian National University, Canberra, ACT Australia; 3 Academic Unit of Ophthalmology, The Australian National University, Canberra, ACT Australia; 4 Research School of Engineering, The Australian National University, and Data61, Commonwealth Scientific and Industrial Research Organisation (CSIRO), Canberra, ACT Australia; 5 School of Information Science and Technology, ShanghaiTech University, Shanghai, China; 6 Research School of Psychology, The Australian National University, Canberra, ACT Australia; 7 Discipline of Optometry and Vision Science, The University of Canberra, Bruce, ACT Australia

## Abstract

Patients with age-related macular degeneration (AMD) have difficulty recognising people’s faces. We tested whether this could be improved using caricaturing: an image enhancement procedure derived from cortical coding in a perceptual ‘face-space’. Caricaturing exaggerates the distinctive ways in which an individual’s face shape differs from the average. We tested 19 AMD-affected eyes (from 12 patients; ages 66–93 years) monocularly, selected to cover the full range of vision loss. Patients rated how different in identity people’s faces appeared when compared in pairs (e.g., two young men, both Caucasian), at four caricature strengths (0, 20, 40, 60% exaggeration). This task gives data reliable enough to analyse statistically at the individual-eye level. All 9 eyes with mild vision loss (acuity ≥ 6/18) showed significant improvement in identity discrimination (higher dissimilarity ratings) with caricaturing. The size of improvement matched that in normal-vision young adults. The caricature benefit became less stable as visual acuity further decreased, but caricaturing was still effective in half the eyes with moderate and severe vision loss (significant improvement in 5 of 10 eyes; at acuities from 6/24 to poorer than <6/360). We conclude caricaturing has the potential to help many AMD patients recognise faces.

## Introduction

Age-related macular degeneration (AMD) is the leading cause of irreversible visual impairment in the developed world^1,2^. It causes central vision loss, reduction in visual acuity, and a visual experience that can include blur, missing parts of the image and distortions^3,4^. As a result, even when vision loss remains mild, AMD impairs recognition of facial identity^5–7^, an ability essential for successful social interactions^8–11^.

**Figure 1:**
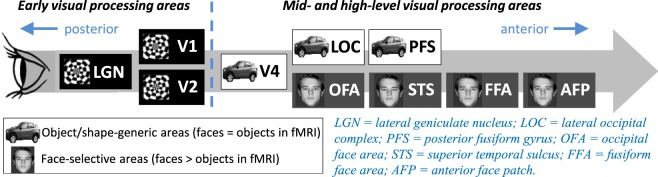
Some of the visual processing areas that respond to faces. Previous image enhancement techniques for improving face identity perception in AMD have targeted low level vision in early visual areas. Our caricaturing method is designed to tap potential for additional benefits from improving coding of face-shape information in mid- and high-level processing regions^17–20^. Note the precise origin of caricature benefits within mid-to-high level regions is not known (although face adaptation aftereffects suggest face perception is influenced by a mix of high-level face-specific coding and more generic opponent shape coding^51^). Image based on Irons *et al*.^33^.

Image enhancement offers potential to improve identity recognition using patients’ remaining vision, for example via digitally enhanced images delivered on computer screen or smart glasses^12,13^. Until now, image enhancement procedures shown to improve face identity perception in AMD have included magnification^5,6^, and increasing the contrast of medium- and high-spatial frequency components in the face image^14–16^. These manipulations are targeted at improving processing in early stages of the visual processing stream (e.g., retina through to V1 and V2). Our approach here is to ask whether there might also be benefit in image enhancement methods that target mid- and/or high-level vision areas where *shape* information is coded^17–20^ (see Fig. 1).

**Figure 2:**
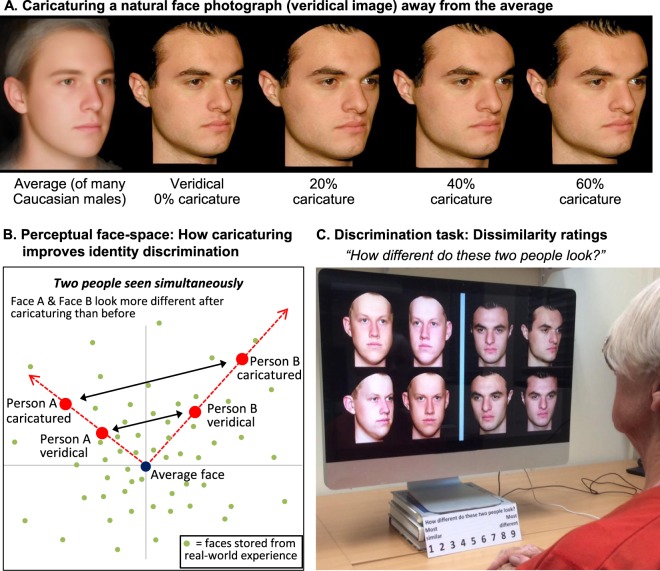
Caricaturing and Experimental Task. (**A**) To make a caricature the veridical face is morphed away from a race/sex/age-matched average, such that all distinctive aspects of the face are exaggerated. In this individual, such aspects include the wide nose, the distance from nose to top lip, the thickness of eyebrows etc. Note that only shape, not colour (which would include lighting information, an unreliable cue to identity) is caricatured in our stimuli. Image based on Irons *et al*.^31^. (**B**) Explanation of caricaturing benefits in terms of a mental face-space. Caricaturing is guaranteed to move any two faces further away from each other in this multidimensional space. Note dimensions coded on the axes remain unknown (but are derived from a participant’s everyday ‘diet’ of faces, and code for both local attributes such as lip thickness and global attributes such as width of the face). Image based on Irons *et al*.^33^. (**C**) Example trial. Faces are shown in 40% caricature strength condition. ‘AMD patient’ played by an actor.

Specifically, we test whether benefits can be obtained from face *caricaturing*. Caricaturing exaggerates the distinctive attributes in a person’s face^21–24^ (Fig. 2A). For example, a man’s natural face (the *veridical* image) may be narrower, have closer together eyes, a larger chin, and a more tilt-tipped nose than the average young adult Caucasian male (the *average face* image). With caricaturing, these differences from the average are exaggerated so that the narrow face becomes even narrower, the close together eyes become even closer together, and so on. Caricaturing of photographic images is achieved using morphing software, which stretches and compresses the distances between corresponding landmark locations (e.g., inner corner of left eye) marked on the target and average faces^21^.

The effects of caricaturing on face perception are explained with reference to a mental face-space^21,22^ (Fig. 2B). In this multidimensional space, faces are coded in terms of how they deviate from the perceptual norm. The norm (average face) and the space’s dimensions are derived from the diet of faces an individual has experienced^25^. Caricaturing corresponds to shifting the face along a trajectory away from the average: staying on this trajectory means the face is still perceived as the same person, but in a more distinctive version of themselves. This makes the face easier to tell apart from all other faces (Fig. 2B) and also improves recognition memory because the density of exemplars in face-space reduces further away from the centre, giving fewer confusable neighbours^21,26^. In addition to caricature effects, there are multiple sources of evidence for the existence of perceptual face-space (including face-antiface adaptation after-effects, better memory for distinctive than typical faces, faster categorisation of typical faces as a face, and density results from multidimensional scaling of pair-wise similarity ratings)^24,26–29^.

Caricaturing improvements in face identification are well established in normal vision for high resolution faces^23,24,30^. More recently, we have applied caricaturing to a partial simulation of AMD — specifically, normal-vision observers shown faces blurred to mimic resolution at different distances into peripheral vision corresponding to AMD disease progression. In these simulated-AMD studies^31,32^ we have shown that caricaturing is effective at improving face recognition accuracy (by approximately 5–14%, using old-new recognition and face-name learning tasks) and increasing perceptual discrimination of identity between faces (i.e., making two faces look more different from each other in a dissimilarity rating task), across a wide range of circumstances. These include young adult observers, older observers (64–86 years) in the age-range relevant for AMD, and multiple levels of blur simulating different levels of vision loss in AMD^31,32^.

In the present study, we provide the first test of caricaturing directly in AMD patients. We assess perceptual discrimination ability for unfamiliar faces, using a dissimilarity rating task^31,33^. In this task (Fig. 2C), observers rate face pairs for how different in identity they appear. Patients were informed there were always two different people, noting this is the situation of practical relevance (i.e., in everyday life, patients know there are two different people in a room because they are in physically different locations). A caricature improvement in identity perception is revealed when ratings increase — indicating the faces look *more* different from each other — as caricature strength is increased (here, across four levels: 0, 20, 40 and 60% exaggeration). We chose this task because, in addition to evidence that its results show good generalisation to recognition tasks (old-new memory, and face-name learning^31,32^), it offers excellent measurement reliability and thus high statistical power^31,33^. This allowed us to evaluate whether caricature improvements were present or absent in individual patients—indeed, individual eyes—rather than merely when averaging over a group. We were then able to efficiently test a wide range of vision loss levels, ranging from extremely mild to legally blind, across 19 AMD-affected eyes (tested monocularly) from 12 patients.

Our research questions were: (a) do caricature improvements in face identity discrimination occur in AMD, (b) if so, are these benefits found only at milder levels of vision loss or do they survive even severe vision loss (e.g., where high-spatial-frequency shape of the internal face features may be completely lost, but lower-spatial frequency information about external face shape, such as breadth across the forehead or chin may still be visible and helped by caricaturing), and (c) how does the size of the mild-vision-loss caricature improvement compare to previous studies of young adults with normal vision^31,33^.

## Method

### Participants and eyes

Participants were 12 AMD patients (8 female, 4 male; age *Mean* = 81.4 years, range 66–93), from whom 19 individual eyes were tested. To be eligible to participate, patients had to: (a) be diagnosed by a qualified ophthalmologist as having AMD in at least one eye (eyes with other diagnoses or without AMD were not tested); (b) not have dementia (and demonstrate good ability to comprehend task instructions); and (c) be Caucasian, to match the race of the face stimuli (and thus avoid poor perception due to the other-race effect^34^).

Recruitment targeted eyes covering the full range of vision loss severity from extremely mild to legally blind (Table 1). Best Corrected Visual Acuity (BCVA) was measured by a qualified orthoptist using a retro-illuminated logMAR chart mounted on a stand conforming to the Early Treatment Diabetic Retinopathy Study (ETDRS) standard format^35^. Additional vision assessment was available for 14 eyes (see Supplement S1).

**Table 1:** Details of eyes, and results.

Eye code (& left or right eye)	Patient code (sex, age)	Visual Acuity BCVA	Diagnosis AMD type	Linear trend statistics	Linear trend *p* score
***Mild***					
E1 (L)	Pa (M,70)	6/6-2	Wet	*F*(1,71) = 32.06, *MSE* = 0.377	<0.001
E2 (R)	Pb (F,88)	6/7.5	Wet	*F*(1,71) = 32.54, *MSE* = 0.506	<0.001
E3 (L)	Pc (F,92)	6/9.5	Wet	*F*(1,71) = 21.01, *MSE* = 0.648	<0.001
E4 (R)	Pd (M,88)	6/9.5	Wet	*F*(1,56) = 12.38, *MSE* = 0.398	0.001
E5 (L)	Pe (M,85)	6/9.5	Wet	*F*(1,71) = 13.94, *MSE* = 1.69	<0.001
E6 (R)	Pf (M,88)	6/12	Wet	*F*(1,71) = 5.41, *MSE* = 0.238	0.023
E7 (L)	Pg (F,76)	6/12	Wet	*F*(1,71) = 19.04, *MSE* = 0.499	<0.001
E8 (L)	Ph (F,78)	6/15	Wet	*F*(1,71) = 5.06, *MSE* = 1.88	0.028
E9 (R)	Pi (F,80)	6/15	Wet	*F*(1,20) = 4.90, *MSE* = 0.543	0.047
***Moderate***					
E10 (R)	Pj (F,73)	6/19	Wet	*F*(1,71) = 0.014, *MSE* = 1.26	0.907
E11 (L)	Pj	6/24	Dry	*F*(1,71) = 0.049, *MSE* = 3.62	0.825
E12 (L)	Pk (F,66)	6/24	Wet	*F*(1,71) = 7.63, *MSE* = 0.275	0.007
E13 (L)	Pd	6/24	Wet	*F*(1,71) = 24.35, *MSE* = 0.676	<0.001
E14 (L)	Pb	6/30	Wet	*F*(1,35) = 12.55, *MSE* = 0.590	0.001
E15 (L)	Pf	6/60	Wet	*F*(1,41) = 1.02, *MSE* = 0.466	0.318
***Severe***					
E16 (L)	Pl (F,93)	6/75	Wet	*F*(1,71) = 4.11, *MSE* = 1.08	0.046
E17 (R)	Pc	6/120	Dry	*F*(1,71) = 1.74, *MSE* = 1.16	0.191
E18 (L)	Pi	<6/360	Wet	*F*(1,20) = 0.015, *MSE* = 0.655	0.905
E19 (R)	Pk	<6/360	Dry	*F*(1,71) = 4.79, *MSE* = 0.488	0.032

Tested eyes ordered by severity of vision loss (best corrected visual acuity), with AMD diagnosis details, statistics for improvement in identity discrimination with caricaturing, and demographics of patient.

*Notes*: Cut-off values for vision loss categories from ICD-10^37^. Visual acuity 6/6-2 indicates patient could read all but 2 letters on the 6/6 line; <6/360 indicates counting fingers only. M = male, F = female. (L) = left eye (i.e., OS, ocular sinister), (R) = right eye (i.e., OD, oculus dextrus). For linear trend statistics, *df* < 71 occurs where patient completed fewer than the full 4 blocks of trials for that eye (see Method). Supplementary Table S1 gives more complete vision data, including for untested eyes.

Recruitment was via The Canberra Hospital Eye Clinic and private ophthalmologist’s rooms, using a study brochure or approach whilst patients were waiting for their consultation. Patients were not paid, beyond reimbursement of travel to the university. Participants gave informed written consent after explanation of the nature and possible consequences of the study. The research methods of the study adhered to the Declaration on Helsinki and were approved by the Australian National University (ANU) and ACT Health Human Research Ethics Committees.

### Task Design and Session Structure

The task was a minor variant on that developed by Irons *et al*.^31^. Two faces were shown simultaneously on the screen, and the 4 images of each person varied in viewpoint and lighting (and thus also in other low-level factors such as specific spatial frequency content); thus patients are being required, deliberately, to rate dissimilarity of the *face* information, not the low-level attributes of the pictures (Fig. 2C). Observers answered the question “How different do these two people look?” on a scale from 1 = most similar to 9 = most different. To ensure *identity*-level face processing was being tested, face pairs were always from the same race/age/sex category: all were Caucasian young adults, and male faces were only ever compared to other males, and females only to other females. Each face pair was repeated across the four strength conditions (0 = uncaricatured veridical face, V; and 20%, 40% and 60% caricature; where 100% indicates doubling distances between landmark points).

Time to test a single eye ranged from 1–3 hours. Total testing time was 2–6 hours per patient (longer durations for patients tested on both eyes), split into sessions of maximum 2 hours conducted on separate days to minimise fatigue.

### Stimuli

Creation of the caricatures is described in full in Irons *et al*.^31,33^. The veridical faces were colour photographs of 26 people (13 male, 13 female), with neutral expression and hair covered. Viewpoints were: frontal; 10° rotation left; 10° rotation right; 30° rotation left (Fig. 2C). Each face was shape-caricatured away from an average face of the same race/age/sex and viewpoint as the target (Fig. 2A), previously created as the average of a large number of individuals of that category (specifically, 57 females and 26 males because our ANU Face Database contains fewer male images; note even 16 faces is sufficient to make a reliable average^36^). Faces were assigned 147 landmark points by hand (or 136 in nonfrontal views showing only one ear).

### Procedure for rating task

For each eye tested, the full experiment comprised four blocks, two showing male faces and two showing female faces. Half the patients did male blocks first, and data were combined across face sex (noting previous evidence face sex does not affect the size of caricature advantages^32^). Within a given face sex, the 13 faces were split into two sets, comprising 7 faces (always used for block 1 of that sex) and 6 faces (for block 2) of different identity.

Within a given block: each face was compared to each other face in the set in turn, always at matched caricature strength (e.g., if Face A was 40% caricature, then Face B was also 40% caricature); each target face appeared on screen-left and screen-right equally often; and each face pairing was shown in each of the 4 caricature strength conditions (intermixed and in different random order for each eye tested). For patients who completed all four blocks of trials for the given eye, this gave 72 trials per caricature strength condition across which to average ratings (i.e., 7 males compared to each of 6 other males = 21 trials, +6 males compared to each of 5 other males = 15 trials, +repeat for females), and a total of 288 trials (i.e., 72 × 4 caricature levels). Across all the patients, 15 of the 19 eyes had complete data for the full four blocks. The other 5 eyes had data for a reduced number of trials (3 blocks for E4, 2 blocks for E14 and E15, 1 block for E9 and E18); this occurred where patients were slow (spending a long time viewing the faces) and/or when they stopped participating for that eye (due to illness, or to not wanting to participate beyond 2 testing sessions total).

Patients were encouraged to use the full range of the rating scale (1 through 9) as much as possible. Instructions (Supplement S2) explained face pairs were always of different people, but the task was to rate, within the upcoming block of faces, which pairs looked *more* or *less* different in identity. Before testing each eye, a demonstration slide showed images of the types of faces they would see (e.g., male young adult Caucasians) and participants practiced pointing to face-pairs they found most similar (and thus might award a low rating), most different (and thus might award a high rating), and pairs that would be rated in the middle of the scale.

Patients viewed the faces for as long as they wished, and gave their rating response verbally; the experimenter entered this using a keyboard. There was a 300 *ms* interval between trials. Patients were monitored for fatigue or discomfort, and offered regular breaks.

### Face size, equipment, viewing distance, free-viewing

Face images were made as large as possible on a 68.5 cm computer screen (Fig. 2C). Average height x width was 14.5 cm × 12 cm (with minor variation due to natural differences in aspect ratio). Target viewing distance was 40 cm, making faces 20.5° x 17.1°. Free viewing was used, to match real world behaviour: there was no chin rest or fixation requirement, and patients could place faces in their best retinal position for viewing by making head rotations, or shifting their head left-right and up-down relative to the screen to best see each of the 8 faces in turn. If patients moved closer to the screen during the experiment, they were asked to move back to the correct viewing distance. Stimuli were presented on an Apple iMac computer (screen resolution = 2560 × 1440 pixels) running OS X, using SuperLab 4.5 software.

### Monocular testing, order of eyes

All testing was monocular, with the other eye covered by an eye patch. Some patients could be tested only on one eye, because: their vision in the other eye was so poor they reported they could not make any judgement about the face pairs; or their other eye did not have AMD. For patients tested on both eyes, we always tested the weaker eye first, to ensure any caricature advantages found with lower-acuity vision could not be attributed to having previously seen the caricatured faces at higher acuity. When subsequently changing to the stronger eye, patients were shown the demonstration slide again to accustom them to how the faces would now appear to them in terms of resolution, and were instructed to recalibrate their use of the rating scale (i.e., noting that faces typically appear more different from each other in absolute terms at higher than lower resolution)^31^.

Testing both eyes separately allowed us to test a wider range of acuity values with the number of patients we had available. Also, in some patients, the monocular testing provided data relevant to whether any failures to show caricature improvements could be attributed specifically to the severity of vision loss in a given eye, as opposed to inadequate cortical coding in the shared brain tapped by both eyes (see Results).

### Statistical analysis

We had sufficient score reliability to perform statistical analysis *within each eye separately*. Some aspects of the analysis differ from statistics averaged over multiple participants. We conducted one-way ANOVA (or more specifically, polynomial trend analysis within ANOVA) *using just the data from the individual eye being analysed*. Because each face pair was repeated across the four caricature levels, statistical analysis on caricature strength (even within a single patient/eye) was repeated measures — but, repeated measures across *items*, not *participants*. Finally, statistical effect size measures (eta-squared) describe proportion of across-*item*-variance explained, rather than the usual proportion of across-*participant*-variance explained, which limits their usefulness (see Supplement S3 for further explanation).

## Results

**Figure 3:**
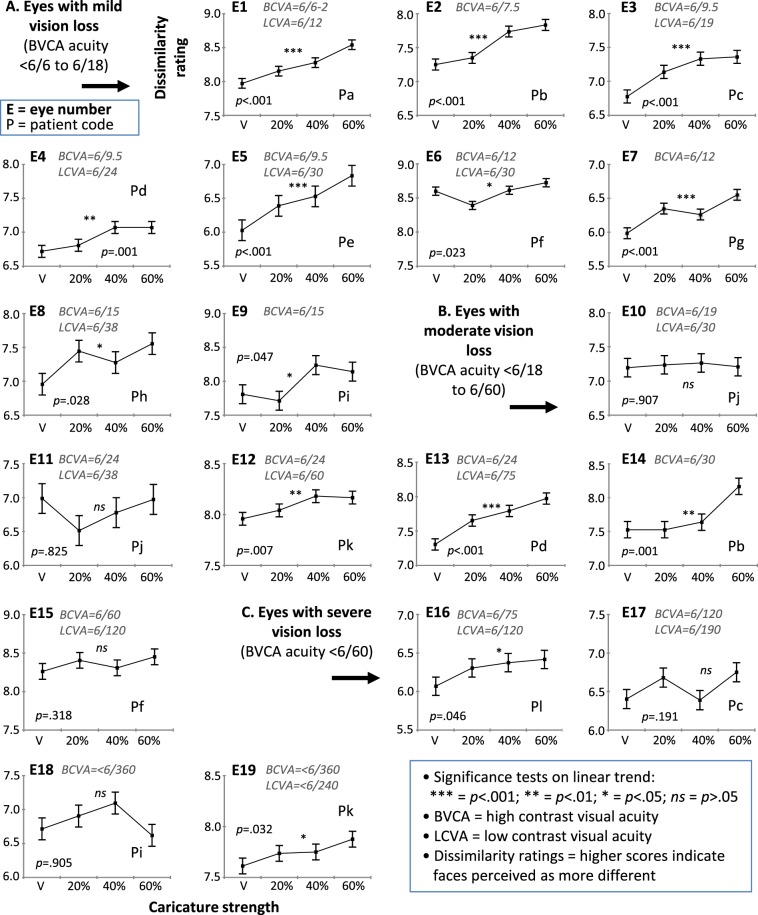
Results for perceived difference in identity between faces, as a function of strength of caricaturing in the face images. Data are shown from 19 eyes (coming from 12 patients) tested monocularly. Eyes ordered from least to most vision loss (top-left to bottom-right). V = veridical (i.e., uncaricatured). Error bars = repeated measures equivalent of SEM. *p* = significance value for linear trend.

Figure 3 plots the perceived difference in identity between faces as a function of strength of caricaturing in the images, separately for each of the 19 individual eyes tested. Eyes are ordered from highest acuity (top left of figure) to lowest (bottom right), and the boundaries for the ICD-10 criteria for mild, moderate and severe vision loss are marked^37^. Pairs of eyes that come from the same patient are indicated by matched participant code. To assist with concrete interpretation of acuity values, poorer than 6/12 (binocularly) results in loss of standard driver’s licence and <6/60 is legally blind (in Australia).

If caricaturing improves identity discrimination, the prediction is that dissimilarity ratings should increase as the strength of caricaturing increases (i.e., the line in each plot should have a positive slope). For each eye separately, we also conducted a statistical test for linear trend; results are in Table 1.

Overall 14 out of the 19 eyes had a significant caricature improvement. This was defined as a linear trend at *p* < 0.05 (two-tailed; in many cases significance level was *p* < 0.01 or *p* < 0.001, Table 1) in the positive direction (Fig. 3).

The benefit of caricaturing was clearest for eyes with mild vision loss. For this category, all 9 eyes—which came from 9 different patients—showed a significant caricature improvement. We also compared the size of this caricature improvement to that shown by young adults with normal vision in previous studies using the same task and stimuli^31,33,38^ (see Supplement S4 for details). For the mild-vision-loss AMD patient group, our mean increase in dissimilarity ratings from Veridical to the maximum 60% caricature strength is 0.504 ± 0.063 (mean ± SE). Comparison values from the three previous young-adult studies were 0.699 ± 0.085 using exactly the same 26 faces seen by the AMD patients, and 0.504 ± 0.090 and 0.684 ± 0.120 using a subset of 20 of the faces; note none of these three differed significantly from the caricature advantage for AMD patients (two-tailed t-tests, all *t*s < 0.005, all *p*s > 0.170). Overall, the size of the caricature improvement in mild vision loss AMD was similar to that shown by young adults with normal vision.

Turning to higher levels of vision loss, caricature improvements were less consistent (Fig. 3, Table 1). Importantly, however, we still found a significant caricature improvement in identity discrimination for half of the eyes in the moderate (3 of 6) and severe (2 of 4) vision loss categories. These included eyes with acuities of 6/24, 6/24, 6/30 and, somewhat remarkably, 6/120 and < 6/360 (i.e., legally blind).

The greater consistency of the caricature improvements for mild vision loss eyes than for the moderate-and-severe vision loss eyes cannot be attributed to any kind of carryover effect from earlier testing of the other, weaker eye (e.g., generalised task practice, or repetition priming due to repeating face items). Table 1 and Fig. 1 demonstrate that, of the 9 mild vision loss eyes, the caricature improvement was as clear in the four cases without prior testing of the other eye (specifically: eye E1 from patient Pa, *p* < 0.001; E5 from Pe, *p* < 0.001; E7 from Pg, *p* < 0.001; E8 from Ph, *p* = 0.028) as it was in the five cases with prior testing (E2 from Pb, *p* < 0.001; E3 from Pc, *p* < 0.001; E4 from Pd, *p* = 0.001; E6 from Pf, *p* = 0.023; E9 from Pi, *p* = 0.047). This argues that it is acuity *per se* that drives the reliability of the caricature improvement.

Similarly, concerning what might explain the *lack* of caricature improvement in the 5 cases where it was absent, results show that in 3 cases (acuities of 6/60, 6/120, and <6/360) this can unambiguously be attributed to the severity of vision loss in the relevant eye: this is because these eyes came from three patients (Pc, Pf and Pi) who were also tested on their other eye which had mild vision loss and showed a significant caricature improvement (Table 1). For the remaining 2 cases, no clear attribution can be made: both these eyes (moderate vision loss, acuities of 6/19 and 6/24) came from the same person (Pj), raising the alternative possibility that this person may have had an inadequate *cortical* coding of faces (i.e., impaired face recognition present before the onset of the AMD, noting poor face-space coding can occur in prosopagnosia^39^). Methodologically, results show the lack of caricature improvements cannot be attributed to reduced statistical power arising from reduced number of trials for some eyes: of the 5 eyes without caricature improvements, 3 received the full number of trials (E10, E11, E17); and, of the 5 eyes that received a reduced number of trials, 3 revealed significant caricature improvements (E4, E9, E14).

Results from two patients also demonstrated that face caricature improvements can survive very poor vision in both eyes, rather than requiring relatively preserved vision (e.g., only mild vision loss) in one eye. These patients are cases where vision loss was moderate-or-severe in *both* eyes, yet a caricature improvement was still observed. Specifically, patient Pk had moderate (6/24) and severe (<6/360) vision loss, and showed a caricature improvement in both these eyes (Table 1). And patient Pl had severe vision loss in both eyes (6/75, and 6/240 in Supplementary Table S1), and showed a caricature improvement in her 6/75 eye (vision in the other eye was too poor for testing).

A final question is what *strength* of caricaturing was most effective. Averaging across the 14 eyes that showed a caricature improvement, perceived difference in identity increased progressively with increasing caricature strength (ratings of 7.18, 7.36, 7.50, 7.66), with 60% caricature strength most effective. (Note strengths higher than 60% are not practical because they produce morphing artefacts in the image and also the faces can begin to look weird and fall outside the neural coding range of face-space dimensions)^40^.

## Discussion

The key results were as follows. All eyes with mild vision loss showed improvement in identity discrimination with caricaturing. In mild vision loss, the size of the caricaturing benefit was as large in AMD patients as in young adults with normal vision. The caricaturing advantage became less stable as visual acuity further decreased. Despite this, caricaturing was still effective in half the AMD cases we tested even with moderate and severe vision loss. Finally, caricature improvements do not require the patient to have one “relatively unaffected” eye (i.e., with only mild vision loss) but can occur even when high quality visual input from either eye has been absent for some time in the patient’s day-to-day experience (which in turn implies the patient’s brain has retained accurate face-space representations despite the lack of recent “topping up” with any high resolution face input). Together, these findings are encouraging for caricaturing as a potential real-world method for improving face identity recognition in AMD, in terms of both breadth of applicability across patients, and the size of the likely caricature improvement.

The rating task used in the present study — selected because of its high reliability allowing individual-eye analysis — does not provide a direct measure of the amount by which caricaturing improves performance accuracy (e.g., accuracy of recognising a person as “Bill”, or as not known). In future studies, it would be valuable to test recognition directly, while noting this would require averaging 30+ participants within a tight range of visual acuity to obtain error bars of acceptable size to determine whether caricature effects are present (at that one acuity level). Importantly, however, there are strong reasons to believe that the presence of caricature improvements in patients’ identity *perception*, as tapped here by dissimilarity ratings, would translate into the presence of caricature improvements in *memory*.

First, in every circumstance our lab has tested to date, we have found that caricature improvements in our dissimilarity rating task translate to caricature improvements in recognition (i.e., old-new memory and/or face-name learning); these circumstances include young adults, older adults, own-race faces, other-race faces, blurred faces, other types of low-resolution faces (a ‘bionic eye’ simulation), and high-resolution faces^31–33^. Second, there are strong theoretical links between caricature improvements in perceiving differences in pairwise identity (our task) and caricature improvements in recognition memory via well-established properties of face-space coding (specifically that exemplar density decreases with increasing distance from the average face^21,26^, which results in fewer nearby confusable neighbours in memory tasks; for review see^22^). Third, we have shown that caricature improvements in memory occur specifically in the age range relevant to AMD: our findings in normal-vision older adults include a faster face-name learning rate, more accurate subsequent recognition of novel images of the learned faces, and no reduction in size of the caricature improvement with increasing age (up to 86 years)^32^. These findings argue that the specific properties of face-space that improve memory remain functional in the brains of older adults of similar age to AMD patients. Overall, given that caricature effects on *perception* remain despite impaired patient *vision* (present article), and caricature effects on *memory* occur in similar-age *brains*^32^, there seems little reason to doubt that the caricature improvements in memory will survive the combination of impaired vision and an elderly brain.

Concerning the likely size of improvement in recognition tasks, a conversion can be made, using the fact that, on dissimilarity ratings, mild-vision-loss AMD patients had a similar level of caricature improvement to young adult observers. Other research from our laboratory^31–33^ using the same face stimuli shows that caricaturing in young adults produces a 5–10% improvement in recognition accuracy, using old-new recognition and face-name learning tasks. Thus, our present results predict a similar performance improvement of 5–10% in mild-vision-loss AMD patients.

An improvement of 5–10% in recognition accuracy in patients is large enough to be of some practical value in the real world. At the same time, it will not get patients back to normal-vision performance. We thus argue that caricaturing should be viewed as a technique that provides useful *incremental* improvement. Caricaturing can be combined with other image enhancement procedures targeting other stages of visual processing of faces (e.g., spatial-frequency-based contrast manipulations^14–16^) that also produce incremental improvements, to produce the greatest total benefit to patients’ functional vision. Translation to patients would then involve potentially several stages of software manipulations to a face image. Note that, currently, computer science is getting fairly close to being able to implement real time caricaturing. Solved steps of the problem include: initial face detection and isolation of the face from the background of a complex natural image^41^; the caricaturing stage itself^23^; and also real-time assignment of landmark points (although in reduced number compared to hand-assignment^42^). There are also some remaining difficulties (e.g., assigning enough landmark points to produce the most accurate caricatures; selecting the correct race/sex/age of average face to caricature away from; see Irons *et al*.^33^ for discussion).

A limitation of the present study is that we test perception of unfamiliar faces only. In future studies, it would be valuable to test whether caricaturing also improves AMD patients’ recognition of pre-experimentally familiar faces, such as friends, family, and famous faces. Of potential relevance to low-vision patients, familiar-face caricature advantages occur in normal vision using impoverished face stimuli^32,43,44^ (although evidence is more mixed for high-resolution faces, see^23,24,45^ vs.^46,47^). Another limitation is that we caricatured only *shape* information in the face. It is possible that caricature benefits for patients could be larger if shape + reflectance information is caricatured, given that reflectance/texture caricaturing can produce performance benefits in normal-vision observers^45,47^ (although note that computing difficulties would need to be overcome to ensure only *identity*-relevant reflectance information was caricatured, not identity-irrelevant lighting information). A final limitation is that we tested patients monocularly, while real world viewing is binocular. In some cases, binocular vision can improve acuity and functional vision^48^. This suggests that perhaps some people with AMD, who have acuity too poor to show a caricature improvement with either eye tested independently, might show a caricature improvement with binocular vision.

Another question for future research is whether caricaturing might be able to improve AMD patients’ perception of information other than faces. A class of wide relevance is *bodies*. Human bodies are used in recognising other people (although less reliably than faces^49^), and, like faces, show evidence of coding relative to an average^50^. It is thus possible that exaggerating an individual’s body shape away from the average may further improve AMD patients’ perception of people’s identity. (Note, however, that caricaturing cannot be applied to basic-level object recognition, e.g., recognition as a dog, tree or mug, because there is no “average object” to exaggerate away from, e.g., dogs, trees and mugs cannot be averaged together).

A final question of interest is whether face caricaturing might be useful in other vision disorders beyond macular degeneration. We predict it will. Across studies, we have shown face caricaturing produces very similar sized benefits for: a blur-only partial simulation of macular degeneration^31,32^; actual AMD patients who commonly experience blur-plus-distortions; and a simulation of prosthetic vision (bionic eye) where images appear as a grid of separated phosphenes of light^33^. Theoretically, this broad generalisability across very different low-resolution formats arises because caricaturing targets high-level visual processing (i.e., cognitive coding of faces). Practically, it implies caricaturing should improve identity perception in any vision disorder that produces low vision.

## Electronic supplementary material

Supplementary Materials

## Data Availability

The de-identified datasets generated during and/or analysed during the current study are available from the corresponding author on reasonable request.
