# Supplementary material for: Improving face identity perception in age-related macular degeneration via caricaturing
Source: Sci Rep. 2018 Oct 12;8:15205. doi: 10.1038/s41598-018-33543-3 (PMC6185956; doi:10.1038/s41598-018-33543-3)
Supplement: Supplementary file 1 — Supplementary Materials [file 41598_2018_33543_MOESM1_ESM.pdf]

SUPPLEMENTARY MATERIALS

Improving face identity perception in age-related macular degeneration via caricaturing

Jo Lane[1], Emilie M. F. Rohan[2], Faran Sabeti[2,7], Rohan W. Essex[3], Ted Maddess[2],

Nick Barnes[4], Xuming He[5], Rachel A. Robbins[6], Tamara Gradden[6], and Elinor McKone[1]

Scientific Reports

[1]Research School of Psychology, and ARC Centre of Excellence in Cognition and its Disorders, The Australian National University, Canberra, ACT, Australia.
[2]John Curtin School of Medical Research (JCSMR), The Australian National University, Canberra, ACT Australia.
[3]Academic Unit of Ophthalmology, The Australian National University, Canberra, ACT, Australia.
[4]Research School of Engineering, The Australian National University, and Data61, Commonwealth Scientific and Industrial Research Organisation (CSIRO), Canberra, ACT, Australia.
[5]School of Information Science and Technology, ShanghaiTech University, Shanghai, China
[6]Research School of Psychology, The Australian National University, Canberra, ACT, Australia.
[7]Discipline of Optometry and Vision Science, The University of Canberra, Bruce, ACT, Australia.

Date: 5 October 2018

Table of contents

• **SUPPLEMENT S1**: More detailed vision information, for both eyes. (Includes Table S1)

• **SUPPLEMENT S2**: Dissimilarity rating task instructions.

• **SUPPLEMENT S3:** Interpretation of proportion-of-variance-explained effect size measures in individual-eye analysis. (Includes Table S2)

• **SUPPLEMENT S4**: Comparison of mild-vision-loss AMD patients to previous experiments in normal-vision young adults. (Includes Table S3)

• **REFERENCES** for Supplementary Materials

SUPPLEMENT S1

More detailed vision information, for both eyes

A more complete vision assessment was conducted for 9 of the 12 patients—covering 14 of the 19 tested eyes—to assess low contrast visual acuity (LCVA) and obtain more detailed retinal information including diagnosis of AMD stage[1]. Full vision testing involved a 90 minute session (which was in addition to the 2-6 hours of face-experiment testing), and was conducted at the Australian National University. Travel reimbursement and ethics/consent was as for the main experiment.

Vision data are shown in Supplementary Table S1. LCVA was measured using a retro-illuminated logMAR chart mounted on a stand conforming to the ETDRS format. Anterior segment of the eye was examined using slit-lamp biomicroscopy, instilling Oxybuprocaine Hydrochloride 0.4% eye drops to anesthetise the eyes to measure intraocular pressure using Goldmann applanation tonometry and to measure central corneal thickness using a Pachmate (DGH Technology Inc., Exton, PA). Patients were tested on 10-2 frequency doubling technology (FDT) threshold using Humphrey Matrix (Carl Zeiss Meditec, Inc., Dublin, CA). After the visual field test both eyes were dilated with Tropicamide 1% and Phenylephrine 2.5% and the following tests were done: Optical Coherence Tomography (OCT) Spectralis (Heidelberg Engineering, Heidelberg, Germany) of the retina (posterior-pole) and the peripapillary retinal nerve fibre layer (pRNFL); scan to measure the thickness of the RNFL surrounding the optic nerve; fundus auto-fluorescence images were acquired, with fundus photography performed using a Canon CR-2 (Canon Inc. Medical Equipment Group, Tokyo, Japan) digital non-mydriatic camera to get an image of the fovea, the macula and the optic nerve. AREDS stages are based on anatomy of the central 6mm of the retina (Stage 1 = Early AMD, small drusen; 2 = Early AMD, intermediate drusen; 3 = Early AMD, large drusen; 4 = covers active exudative, choroidal neovascularisation for Wet AMD, and

end-stage Dry AMD/sub-foveal geographic atrophy. For Stages 1-3 visual acuity is usually close to normal; for Stage 4, acuity can vary widely between normal and <6/60 (legally blind), e.g., depending on treatment (for Wet AMD).

Supplementary Table S1. Patient vision information for both eyes.

Patient code	Eye code (left or right)	Visual Acuity BCVA	LCVA	Diagnosis AMD type	AREDS Stage
Pa	E1 (L)	6/6-2#	6/12	Wet AMD	2
	n/a (R)	6/190	<6/240	Corneal scar, amblyopia/No AMD	n/a
Pb	E2 (R)	6/7.5	–	Wet AMD	–
	E14 (L)	6/30	–	Wet AMD	–
Pc	E3 (L)	6/9.5	6/19	Wet AMD	4
	E17 (R)	6/120	6/190	End-stage AMD/Dry	4
Pd	E4 (R)	6/9.5	6/24	Wet AMD	4
	E13 (L)	6/24	6/75	Wet AMD	4
Pe	E5 (L)	6/9.5	6/30	Wet AMD	4
	n/a (R)	6/7.5	6/12	Vitrectomy/No AMD	n/a
Pf	E6 (R)	6/12	6/30	Wet AMD	4
	E15 (L)	6/60	6/120	Wet AMD	4
Pg	E7 (L)	6/12	–	Wet AMD	–
	n/a (R)	6/6	–	No AMD	n/a
Ph	E8 (L)	6/15	6/38	Wet AMD	4
	n/a (R)	6/190	<6/240	Wet AMD	4
Pi	E9 (R)	6/15	–	Wet AMD	–
	E18 (L)	<6/360	–	Wet AMD	–
Pj	E10 (R)	6/19	6/30	Wet AMD	4
	E11 (L)	6/24	6/38	Early AMD/Dry	3
Pk	E12 (L)	6/24	6/60	Wet AMD	4
	E19 (R)	<6/360	<6/240	End-stage AMD/Dry	4
Pl	E16 (L)	6/75	6/120	Wet AMD	4
	n/a (R)	6/240	<6/240	End-stage AMD/Dry	4

Notes: LCVA = low contrast visual acuity; LCVA <6/240 indicates the patient could not read all letters on the largest line of the LCVA chart. LCVA correlated very highly with BCVA (*r*=.96 for 14 eyes with LCVA scores). Patients Pb, Pg and Pi did not have a vision assessment at the ANU; BCVA and diagnosis were provided by their ophthalmologist. Patients Ph and Pl had AMD in their weaker eye, making the eye eligible for the study, but with this eye reported they could not see the faces well enough to rate them (e.g., because images were too blurred and/or they could not see major parts of the face) and therefore this eye was not tested. n/a = eye not tested or not eligible. For additional notes see Table 1.

SUPPLEMENT S2

Dissimilarity Rating Task Instructions

Key sections from the Experimenter Script:

FIRST EYE TO BE TESTED (weaker eye if both tested)

• You will be looking at faces on the computer screen and making some decisions about them. *Place the participant 40 cm from the screen.* You are free to move your head around when you are looking at the screen, if you find that moving the location of your head helps you see the faces more clearly. Please don't move your face forward, closer to the screen. If I notice you are moving forward during the experiment, I will place you back to the correct position.

• At any time during the experiment if you need to move, stand up, stretch or have a break please let me know. Also, if you are finding the task tiring, or straining on your eyes, let me know and we can take a break. *Check the participant is in a comfortable position e.g. chair height etc.*

• You will see images like this one (*image of task on the computer screen*) that have four photos of one person on the left side of the screen, a line down the middle, and four photos of a different person on the right side of the screen. You can see here on the left side of the screen (*point*), there are four different photos. These are all photos of one person that are taken at four different angles so you can get an overall look at that person's face. Here on the right side of the screen (*point*) is another person, again with four photos taken at four different angles so you can get an overall look at that person's face. Does that make sense?

• I'll be showing you different pairs of people on each trial and what I want you to do is tell me **how different the two people look to you**, on a 9-point scale ranging from "Most similar" to "Most different" by choosing a number between 1 and 9 (*point to hard copy of scale below the computer screen*).

• We would like you to make your judgment based on each person's face, not just what a particular photograph of them looks like. So please try to focus on how different the two people appear to you, rather than on how different some superficial aspect of the images appears e.g., the size of the photo, lighting in the photo or the colour tone of the photo.

• You need to select a number between 1 and 9 and say it out loud and I will enter your response on the keyboard.

• When you rate how different the two faces on the computer screen look, you need to make your judgments based on how different the faces look relative to each other within the set of faces. For example, you would respond with the number 1 if you thought the two male faces on the screen are the MOST SIMILAR compared to the male faces that you saw in that block of male faces.

• Or if you were comparing female faces, you would press the number 9 if you thought the two female faces on the screen are the MOST DIFFERENT compared to the female faces that you saw in that block of female faces.

• For each eye you will be presented with four blocks of faces, two blocks of female faces and two blocks of male faces.

• In the first block of the experiment you will be using your weaker eye and your stronger eye will be covered with an eye patch. This will be reversed in the second block.

• Please cover your stronger eye now so you are only using your weaker eye.

Your eye may take a little time to adjust. *Wait for participant to say their weaker eye has adjusted.*

• Please use the full range of the 1 to 9 scale so we can see the range of differences between the faces within the set. The next slide will show you some of the male/female faces you will see in the male/female block and how much the male/female faces vary.

• To help you get an idea of the task and work out how you might use the rating scale, here is a slide that has six different male/female faces that you will see during the experiment. Here you can see the variation in the different faces you will see. As you might be able to see, the faces are all adults, all young, all white Caucasian and don't vary much in hairstyle because we have hidden most of their hair. Can you see the faces on the screen? Can you tell that the photos are all of different people?

• Looking at these faces, can you point to two faces that look MOST SIMILAR TO EACH OTHER WITHIN THESE MALE/FEMALE FACES? Using the rating scale (*pointing to it*) what number on the rating scale might you give if you saw those two faces come up together during the experiment? (*Should say they would respond with a low number, e.g., 1 or 2*)

• Looking at these faces, can you point to two faces that look MOST DIFFERENT FROM EACH OTHER WITHIN THESE MALE/FEMALE FACES? Using the rating scale (*pointing to it*) what number on the rating scale might you give if you saw those two faces come up together during the experiment? (*Should say they would respond with a high number, e.g., 8 or 9*)

• Can you point to two faces that you think would fall in the MIDDLE of the scale (e.g., you would respond to with a 4, 5 or 6)?.

• Do you have any questions about the experiment? Please get comfortable (check seating position). Let me know when you are ready to begin the experiment.

• *Between blocks have a break e.g., stretch, tea/coffee etc.*

IF SECOND EYE TO BE TESTED (which would be the stronger of the two)

• The task you have to do is exactly the same as before, however this time you will be using your stronger eye and your weaker eye will be covered with an eye patch.

• Like before, your task in this block is to indicate **how different** the two people's faces look on a 9-point scale ranging from "1 = Most similar" to "9 = Most different" within the set of faces.

• The only difference in this block is that you may find the way you use the scale is different because you are using your stronger eye. For example, you may notice the differences between the faces within a set more easily now because you can see the differences more clearly. This is to be expected.

• You need to completely change how you assign the scale numbers compared to the first half of the experiment. Base your judgment on the way the faces look to you now, not as they did with your weaker eye.

• Again, your task is to rate how different the faces look and make your judgments based on how different the faces look relative to each other within the set of faces

e.g., 1 = "most similar within this set of males". The next slide will show you some of the male/female faces you will see in the male/female block and how much the male/female faces vary.

• Please cover your weaker eye now so you are only using your stronger eye. Your eye may take a little time to adjust.

• *Rest of instructions as for first eye.*

SUPPLEMENT S3

Interpretation of proportion-of-variance-explained effect size measures in individual-eye analysis

As noted in the main-text Methods, effect size measures such as eta-squared mean something quite different in our individual-eye analysis (i.e., specifically proportion of across-*item*-variance explained), as compared to the more common situation where scores are averaged over participants (i.e., proportion of across-*participant*-variance explained).

The logic behind the usual interpretation of effect size measures is that the type of variance being explained is meaningful — that is, in the case of analysis averaging over participants, the standard argument is that variation between different people is meaningful and that one wishes to explain this. Thus, for example, saying that sex explains 4% of variance in mathematics test scores would be interpreted as meaningful evidence of a small effect (i.e., because it would indicate that the mean difference between males and females was small compared to the overall variability in peoples' maths ability).

However, in our case of individual-eye analysis, effect size measures (eta-squared) describe the proportion of variance in ratings for different *items* (i.e., specifically, the different face pairings) that can be explained by caricaturing. In absolute terms, this is not a meaningful measure. For example, if we find that 8% of variance in a person's face-pair dissimilarity ratings can be attributed to caricaturing, the 8% value per se is meaningless: had we selected a different set of 26 faces, or paired them up differently (e.g., so that some pairs were more different, or less different, in appearance than within our current pairings), then we could have obtained a completely different value (i.e., simply because the *variance* value will change, not the actual caricature impact).

This limits the usefulness of effect size measures in our design to *relative* comparisons where the items are identical across the situations compared. For example, it is

valid to ask whether the *statistical effect size correlates with acuity* for the 14 eyes tested *on an identical item set* (i.e., the 14 eyes tested on all four blocks and thus all 72 face pairs). These 14 eyes are listed in Table S2, and show a significant correlation between greater vision loss (acuity coded as logMAR) and reduced proportion of across-item-variance explained by caricaturing, $r = -.572$, $p = .033$. Note the table and correlation calculation excludes the 5 eyes for which not all items were tested (e.g., they may have completed only the female-face blocks and not the male-face blocks, see Methods); this is because variance across a smaller set of items cannot be validly compared to variance across a different, larger set of items.

Table S2. Effect size: Eta-squared for the linear trend on the caricature effect (for the 14 eyes tested on all 72 items) against acuity.

Eye	Patient	Acuity (BCVA)	Acuity expressed as logMAR	Linear trend partial eta-sq
E1	Pa	6/6-2	0.04	.311
E2	Pb	6/7.5	0.1	.314
E3	Pc	6/9.5	0.2	.228
E5	Pe	6/9.5	0.2	.164
E6	Pf	6/12	0.3	.071
E7	Pg	6/12	0.3	.211
E8	Ph	6/15	0.4	.067
E10	Pj	6/19	0.5	.000
E11	Pj	6/24	0.6	.001
E12	Pk	6/24	0.6	.097
E13	Pd	6/24	0.6	.255
E16	Pl	6/75	1.1	.055
E17	Pc	6/120	1.3	.024
E19	Pk	<6/360	1.8	.063

SUPPLEMENT S4

Comparison of mild-vision-loss AMD patients to previous experiments in normal-vision young adults

As described in main text Results, we compared the amount of caricature improvement (difference between rating for 60% Caricature and rating for Veridical faces) in the mild-vision-loss AMD patient group to caricature improvements in the same rating task in three previous experiments that used young adults with normal vision. Means for Veridical and 60% Caricature separately from these experiments are shown in Supplementary Table S3. Also, key features and publication details of these previous experiments are:

• Study 1. Experiment 1 of Irons et al. (2014). This experiment used a subset of 20 of the present 26 faces. It also tested conditions not reported here (with trials intermixed with the reported conditions), including intermediate 20% and 40% caricature strengths, and 3 blur levels; data in Table S3 are for high resolution (i.e., unblurred) faces. The published experiment reported data for N=12 participants; we also later tested an additional N=10 participants on exactly the same experiment. All participants were Caucasian (same race as the face stimuli, and AMD patients).

• Study 2. Experiment 1 of Irons et al. (2017). This experiment used a subset of 20 of the present 26 faces. It also tested conditions not reported here (with trials intermixed with the reported conditions), including an intermediate 40% caricature strength, and a bionic eye simulation condition (40x40 phosphene grid); the published data in Table S3 are for high resolution faces. All participants were Caucasian.

• Study 3. Experiment 1 of McKone et al. (in press). This experiment used all 26 of the present faces (paired exactly as here, i.e., grouped into same set of 7 and 6 of each sex). It also tested conditions not reported here (with trials intermixed with the reported conditions), including 2 blur levels and a condition where caricatures were made using fewer landmark

points; data in Table S3 are for high resolution (i.e., unblurred) faces and for the same 147-point caricatures as used for the AMD patients. All participants were Caucasian.

Table S3. AMD patients and previous studies of young adults with normal vision.

		Veridical		60% Caricature		Improvement (60%-V)	
**Study & participants**	**N**	**Mean**	**SEM**	**Mean**	**SEM**	**Mean**	**SEM**
Present study, mild-vision-loss AMD patients	9	7.112	.276	7.626	.235	.504	.063
Young adults Study 1 [Irons et al. 2014]	12	6.408	.169	6.667	.185	.504	.090
- additional participants on same experiment	10	6.110	.344	6.425	.315	.558	.139
Young adults Study 2 [Irons et al. 2017]	20	6.109	.257	6.793	.235	.684	.120
Young adults Study 3 [McKone et al. in press]	20	5.358	.158	6.057	.122	.699	.085

REFERENCES FOR SUPPLEMENTARY MATERIALS

1 Age-Related Eye Disease Study (AREDS) Research Group. The Age-Related Eye Disease Study system for classifying age-related macular degeneration from stereoscopic color fundus photographs: the Age-Related Eye Disease Study Report Number 6. *Am J Ophthalmol.* **132,** 668-681, doi: 10.1016/S0002-9394(01)01218-1 (2001).

2 Irons, J. L. *et al.* A new theoretical approach to improving face recognition in disorders of central vision: Face caricaturing. *J Vis.* **14,** 1-29, doi :10.1167/14.2.12 (2014).

3 Irons, J. L. *et al.* Face identity recognition in simulated prosthetic vision is poorer than previously reported and can be improved by caricaturing. *Vision Res*. **137,** 61-79, doi: 10.1016/j.visres.2017.06.002 (2017).

4 McKone E., Robbins R.A., He X. & Barnes N. Caricaturing faces to improve identity recognition in low vision simulations: How effective is current-generation automatic assignment of landmark points? *PLoS ONE.* (in press).
